# Additional considerations for "checklists to guide the supportive and critical care of tuberculous meningitis"

**DOI:** 10.12688/wellcomeopenres.15749.1

**Published:** 2020-04-06

**Authors:** Anuradha Behl, Sumeet Dhawan

**Affiliations:** 1 Department of Pediatrics, Maharishi Markandeshwar Institute of Medical Sciences and Research (Deemed to be University), Mullana, Ambala, Haryana, 133207, India

**Keywords:** TBM, meningeal tuberculosis, chronic meningitis, reporting guidelines, tuberculosis, tubercular meningitis

## Abstract

Checklists are pivotal in the systematic assessment of critically ill patients, pre-operative assessments and for patients with multisystem involvements. Management of tuberculous meningitis is challenging due to prolonged hospital stay, multiple neurological complications like seizures, stroke, raised intracranial tension, stroke, neurosurgical interventions, multiple invasive procedures, health-care-associated sepsis, and ventilation. All these complications are managed by separate checklists to avoid treatment-related errors. The current manuscript aims to ensure completeness of inpatient care addressing issues addressing diagnostic issues, supportive care, and intensive care related issues.

## Correspondence

We read the article by Donovan *et al.* with great interest ^1^. We suggest including certain additional details in the checklist and tables provided, as we describe in Table 1–4 ^2, 3^. These details are vital to ensuring quality supportive care in the daily assessments of patients with tuberculous meningitis.

**Table 1.** Additional considerations in the supportive care checklist for tuberculous meningitis.

Table / sub-section	Additional considerations
Table 1 – Initial evaluation	
History	• History of recent TB-positive contacts: smear-positive or negative, regimen used, compliance, intermittent or daily regimen
General clinical examination	• Weight and nutritional status (and head circumference in children)
Neurological examination	• Assessment of fundus for choroidal tubercles
Laboratory tests (CSF)	• Other CSF analyses like fungal smear, cryptococcal antigen, and India ink
Imaging	• Ultrasound of abdomen to check for lymphadenopathy • Chest radiograph of symptomatic family contacts to detect early forms of tuberculosis
Table 2 - Daily inpatient review	
General clinical examination	• Position change two to three times hourly to prevent bedsores
Medication evaluation	• Confirm the dosage and strength of ATT medications advised • Collect blood culture reports and plan duration/de-escalation of antibiotics (e.g., third-generation cephalosporin) and level of ulcer prophylaxis
Neurological examination	• Does the patient need to repeat neuroimaging? A repeat CT scan is advisable within six hours of a neurosurgical intervention like a ventriculoperitoneal shunt if there is no improvement in neurological status, and after 3–7 days (if not done earlier) to check the position of the ventriculoperitoneal shunt with low radiation protocol
Table 3 - Critical care	
General clinical examination	• Examination of bedsores
Vascular access	• Is intravenous cannula still needed? • Examination of sites of vascular access (intra-venous, central) for change in dressing, dislodgement, etc.
Table 4 - Priorities checklist for the acutely deteriorating patient with TBM	
Reduced consciousness	• Consider liver function test and ammonia levels for hepatitis • Monitoring drug (antiepileptic) levels for drug toxicity • Have seizures been excluded? Consider bedside EEG to exclude nonconvulsive status epilepticus • Exclude mechanical factors like displacement of the endotracheal tube, break in oxygen supply

TB, tuberculosis; TBM, tuberculous meningitis; CSF, cerebrospinal fluid; ATT, anti-tubercular treatment; CT, computed tomography; EEG, electroencephalogram.

### Recommended additions to Table 1

The assessment of clinical records of contacts of tuberculosis (TB) patients provides valuable details regarding the likelihood of resistance, with 7% of TB patients likely to have an undiagnosed family member with TB ^4^. This is particularly vital as isolation rates for acid-fast bacilli (AFB) in tuberculous meningitis are low, and proving resistance is difficult ^5^. The undiagnosed contact may provide an early clue to drug sensitivity, as the sensitivity of AFB isolation is higher from sputum samples compared to cerebrospinal fluid ^6, 7^. Visual identification of choroidal tubercles may increase the likelihood of tuberculous meningitis in a patient with meningitis (especially if AFB smear is non-contributory) ^8^.

Another missed clinical detail is whether TB-positive contacts have completed treatment and been declared cured. Often treatment is completed, but confirmation of having been cured is not documented by repeat AFB smears at five months (or end) of treatment ^9, 10^. The World Health Organization (WHO) suggests AFB smear testing at diagnosis, two months (or three months if the intensive phase is extended by one month), and five months of treatment. However, simultaneous culture should also be performed, as a sub-group of patients may have only AFB smear positivity (with no clinical progression) and negative cultures due to the presence of non-viable AFB ^9^. We suggest chest radiography of all members for contact screening in family members. In one study, 13% of household contacts had abnormal chest X-rays, out of which 30% were asymptomatic ^11^. This may be particularly useful in probable tuberculous meningitis patients (AFB smear negative). Similarly, ultrasonography of the abdomen may provide ancillary evidence of tuberculosis and provide additional tissue (especially lymph nodes) for isolation of AFB ^12^. Patients with chronic meningitis may have similar clinical and radiological features. Therefore, all patients should be evaluated for mimickers such as non-tuberculous mycobacteria, cryptococcal meningitis, fungal meningitis, carncinomatous meningitis, neurosarcoidosis, collagen vascular disease, toxoplasmosis and cytomegalovirus depending on the epidemiology ^13^.

In daily inpatient review, daily monitoring of head circumference (in infants) provides an early clue for worsening hydrocephalous, and periodic ultrasonography of the head in infants helps in the safe monitoring of hydrocephalous ^14^.

### Recommended additions to Table 2

Multiple fixed-dose combinations and various strengths of individual drugs of anti-tuberculous drugs are available. Not uncommonly, errors in checks carried out by physicians or pharmacists lead to over or under-dosage, especially in children. To reduce prescription errors for dosages, dosing charts based on weights with standard fixed-dose combinations have been suggested by the WHO ^15^. In an individual situation, one or more drugs need to be given separately to refine the treatment. Therefore, we suggest that the total dose of individual medications be calculated and separately considered in daily inpatient evaluation to prevent drug failure or toxicity. Post-operative cranial tomography is usually done in the post-operative period to assess the position of a ventriculoperitoneal shunt and reduction in the size of the ventricle. We suggest a low radiation protocol (40 mAs, 120 kV) for such imaging procedures, which may reduce radiation exposure by 90% ^16^.

### Recommended additions to Table 3

Prolonged immobility due to chronic encephalopathy predisposes patients to pressure sores. Daily assessment of indwelling catheters is vital to prevent health-care-associated infections ^17^.

### Recommended additions to Table 4

Treatment-related complications such as drug-induced hepatitis, anti-tuberculous therapy-induced psychosis, and phenytoin toxicity should be excluded as a cause of reduced consciousness ^18, 19^. Anti-tubercular drugs like isoniazid, rifampicin, ethambutol, and cycloserine may be associated with psychosis ^20, 21^. Isoniazid-associated psychosis may occur as early as three days to as long as several months after initiation of isoniazid. These symptoms may manifest as delirium, delusions, suicidal tendencies, and mood swings ^19^. Though complications of tuberculous meningitis, such as infarct, borderzone encephalitis, and hydrocephalus, can lead to encephalopathy, bedside electroencephalography should be done in all such patients to exclude nonconvulsive status epilepticus ^22^. Acute symptomatic seizures are frequent in tuberculous meningitis, and antiepileptic drug levels such as phenytoin, phenobarbitone, levetiracetam, sodium valproate are commonly used drugs. Anti-tubercular drugs have complex drug interactions amongst themselves, along with other medications ^23^. Isoniazid and valproate are drug inhibitors, while rifampin, phenobarbitone phenytoin are drug inducers. The drug levels of isoniazid depend on acetylation status ^24^. Patients who are slow acetylators have higher isoniazid concentration, lower acetylated-isoniazid, and higher phenytoin concentrations. These patients are at high risk of phenytoin toxicity and encephalopathy ^24^. The authors observed that one-third of patients with encephalopathy had higher phenytoin drug concentration ^24^. Though levetiracetam is devoid of drug interactions, it may also cause behavioral abnormalities, including psychosis and suicidal tendencies ^25^.

## Data availability

No data are associated with this article.
