# Supplementary material for: Additional considerations for "checklists to guide the supportive and critical care of tuberculous meningitis"
Source: Wellcome Open Res. 2020 Apr 6;5:64. [Version 1] doi: 10.12688/wellcomeopenres.15749.1 (PMC7405257; doi:10.12688/wellcomeopenres.15749.1)
Supplement: Peer review history

# Peer review: Reviewer response for version 1

**DOI:** 10.21956/wellcomeopenres.17268.r39346

**Published:** 2020-08-05

**Authors:** Anju Gupta, Arushi Gahlot Saini

**Affiliations:** 1 Department of Pediatrics, Postgraduate Institute of Medical Education and Research (PGIMER), Chandigarh, Punjab and Haryana, India; 2 Postgraduate Institute of Medical Education and Research (PGIMER), Chandigarh, Punjab and Haryana, India

The authors have suggested useful additions to the checklist. We have the following suggestions:

 History: Although it seems ideal to ask for ‘smear-positive or negative, regimen used, compliance, intermittent or daily regimen’, it is often difficult in the developing countries where the majority of the population is from rural background and record keeping is not adequate. Hence, the least that should be asked is:

- If at all they were diagnosed by TB and of which site
- Duration of treatment
- Whether family members, esp. children were screened or put on prophylaxi
- Were they put on any drugs that had to be consumed daily or alternate day in the morning and causes change in the colour of urine
- Were they affiliated to any DOTS centre?

 General clinical examination: Weight and nutritional status (and head circumference in children).

 The head circumference has already been mentioned by the primary authors in the section on neurological examination, so it is not really an addition

 The examination should also include:

- Evaluation of respiratory system for parenchymal or pleural pathology
- Evaluation of cardiac system for pericardial pathology
- Evaluation for meningismus: it is especially important in patients with altered sensorium
- Evaluation of spine for gibbus, kyphosis or any paravertebral masses
- Evaluation for a neurogenic pattern of breathing
- Chronic non-healing ulcers, chronic discharging sinuses in the skin, pathological fractures, spina ventosa which are often the initial peripheral sings and wrongly attributed to trauma
- Evolution of new focal deficits suggestive of vascular complications

Daily inpatient review:

- Medication: Addition of pyridoxine with INH to prevent peripheral neuropathy
- Nutritional status, especially protein intake in the diet and vitamin deficiencies, including B12, D and K

Are arguments sufficiently supported by evidence from the published literature or by new data and results?

Yes

Is the conclusion balanced and justified on the basis of the presented arguments?

Yes

Is the rationale for commenting on the previous publication clearly described?

Partly

Are any opinions stated well-argued, clear and cogent?

Yes

Reviewer Expertise:

clinical general pediatrics, pediatric rheumatology and immunodeficiency

We confirm that we have read this submission and believe that we have an appropriate level of expertise to confirm that it is of an acceptable scientific standard.

# Peer review: Reviewer response for version 1

**DOI:** 10.21956/wellcomeopenres.17268.r39347

**Published:** 2020-08-04

**Authors:** Anne M. Doherty

**Affiliations:** 1 University College Dublin, Dublin, Ireland; 2 Mater Misericordiae University Hospital, Dublin, Ireland

In this commentary, Behl and Dhawan suggest additional factors to be considered in the management of tuberculous meningitis, focussing on the neurological and neuropsychiatric sequelae of both the condition itself and of medications used - in particular the suggested additions to Tables 2 and 4 are relevant and clinically important.

 The point regarding contact tracing is well made.

Are arguments sufficiently supported by evidence from the published literature or by new data and results?

Yes

Is the conclusion balanced and justified on the basis of the presented arguments?

Yes

Is the rationale for commenting on the previous publication clearly described?

Yes

Are any opinions stated well-argued, clear and cogent?

Yes

Reviewer Expertise:

Liaison psychiatry (which includes the neuropsychiatric manifestations of physical illness)

I confirm that I have read this submission and believe that I have an appropriate level of expertise to confirm that it is of an acceptable scientific standard.

# Peer review: Reviewer response for version 1

**DOI:** 10.21956/wellcomeopenres.17268.r38369

**Published:** 2020-05-18

**Authors:** Suvasini Sharma

**Affiliations:** 1 Neurology Division, Department of Pediatrics, Lady Hardinge Medical College and Associated Kalawati Saran Children's Hospital, New Delhi, Delhi, India

The authors have suggested useful additions to the checklist. In clinical practice, apart from the disease and the anti-tubercular treatment, effects and interactions with co-medications such as anti-epileptic drugs must be considered. Seizures are common in the acute phase of TBM. I would also suggest addition of fever, in the general condition. Though temperature is mentioned, the trend of fever (reduced peaks, duration etc) in the last 24 hours is also an important parameter to assess improvement, or deterioration.

Are arguments sufficiently supported by evidence from the published literature or by new data and results?

Yes

Is the conclusion balanced and justified on the basis of the presented arguments?

Yes

Is the rationale for commenting on the previous publication clearly described?

Yes

Are any opinions stated well-argued, clear and cogent?

Yes

Reviewer Expertise:

Epilepsy, Neuroinfections

I confirm that I have read this submission and believe that I have an appropriate level of expertise to confirm that it is of an acceptable scientific standard.
